# Supplementary material for: Comparative Effects of Abdominal Bracing and Valsalva Maneuver on Cerebral and Peripheral Hemodynamics in Healthy Adults: A Randomized Crossover Study
Source: Medicina (Kaunas). 2025 Nov 14;61(11):2031. doi: 10.3390/medicina61112031 (PMC12654084; doi:10.3390/medicina61112031)
Supplement: Supplementary file 1 [file medicina-61-02031-s001.zip › medicina-3929549-supplementary.pdf]

medicina

MDPI

Table S1. Comparison of heart rate and peripheral capillary oxygen saturation responses between the Valsalva maneuver and abdominal bracing.

			Repeated-measures comparison			Post hoc comparison (Tukey)			
**Group**	**Parameter**	**Variable**	**Mean ± SD**	**F**	***p***	**Repeated-measures factor**		**t**	***p*Tukey**
VM	HR	Pre	70.57 ± 9.13	9.28	< 0.001*	Pre	Post1	–3.73	0.007*
							Post2	0.12	1.000
		Post1	75.86 ± 10.96				Post3	0.62	0.971
							Post4	0.85	0.911
		Post2	70.46 ± 7.34			Post1	Post2	3.75	0.006*
							Post3	3.55	0.011*
		Post3	70.10 ± 9.60				Post4	3.36	0.017*
						Post2	Post3	0.45	0.991
		Post4	69.93 ± 9.56				Post4	0.53	0.983
						Post3	Post4	0.35	0.997
	SpO2	Pre	97.67 ± 1.01	0.02	0.991	Pre	Post1	–0.17	1.000
							Post2	–0.25	0.999
		Post1	97.73 ± 1.31				Post3	–0.26	0.999
							Post4	–0.40	0.994
		Post2	97.75 ± 1.16			Post1	Post2	–0.05	1.000
							Post3	0.02	1.000
		Post3	97.75 ± 1.11				Post4	–0.01	1.000
						Post2	Post3	0.08	1.000
		Post4	97.74 ± 0.98				Post4	0.03	1.000
						Post3	Post4	–0.07	1.000
AB	HR	Pre	69.05 ± 9.53	20.27	< 0.001*	Pre	Post1	–5.08	< 0.001*
							Post2	–4.12	0.002*
		Post1	80.33 ± 14.44				Post3	–1.45	0.600
							Post4	0.40	0.994
		Post2	74.75 ± 9.49			Post1	Post2	3.96	0.004*
							Post3	4.84	< 0.001*
		Post3	70.40 ± 8.47				Post4	4.90	< 0.001*
						Post2	Post3	4.15	0.002*
		Post4	68.70 ± 9.55				Post4	4.09	0.003*
						Post3	Post4	2.72	0.075
	SpO2	Pre	98.43 ± 0.71	5.56	<0.001*	Pre	Post1	3.17	0.027*
							Post2	2.68	0.082
		Post1	97.49 ± 1.76				Post3	1.37	0.650
							Post4	1.07	0.819
		Post2	97.68 ± 1.45			Post1	Post2	–0.71	0.954
							Post3	–2.47	0.125
		Post3	98.30 ± 0.75				Post4	–2.52	0.113
						Post2	Post3	–2.15	0.227
		Post4	98.30 ± 0.75				Post4	–2.21	0.205
						Post3	Post4	–0.08	1.000

Abbreviations: VM, Valsalva maneuver; AB, abdominal bracing; HR, heart rate; SpO_2, peripheral capillary oxygen saturation; SD, standard deviation; * $p < 0.05$.

Table S2. Comparison of blood pressure responses (systolic and diastolic) during the Valsalva maneuver and abdominal bracing.

Group	Parameter	Variable	Mean ± SD	$Mean_{diff} \pm SE_{diff}$	t	*p*
VM	SBP	Pre	110.23 ± 9.46	−1.333 ± 0.515	−2.59	0.015*
		Post	111.57 ± 10.33			
	DBP	Pre	70.97 ± 6.12	−0.767 ± 0.392	−1.96	0.060
		Post	71.73 ± 6.48			
AB	SBP	Pre	109.77 ± 9.62	−2.033 ± 0.478	−4.25	< 0.001*
		Post	111.80 ± 9.88			
	DBP	Pre	70.63 ± 6.81	−1.633 ± 0.344	−4.75	< 0.001*
		Post	72.27 ± 7.01			

Abbreviations: VM, Valsalva maneuver; AB, abdominal bracing; SBP, systolic blood pressure; DBP, diastolic blood pressure; SD, standard deviation; SE_{diff}, standard error differences; * $p < 0.05$.

Table S3. Comparison of vascular hemodynamic responses (pulsatility index and resistive index) during the Valsalva maneuver and abdominal bracing.

		Repeated-measures comparison				Post hoc comparison (Tukey)			
Group	Parameter	Variable	Mean ± SD	F	*p*	Repeated-measures factor		t	p_{Tukey}
VM	PI	Pre	1.76 ± 0.28	4.48	0.008*	Pre	Post1	−1.67	0.468
							Post2	−3.29	0.020*
		Post1	1.82 ± 0.33				Post3	−2.65	0.087
							Post4	−1.74	0.428
		Post2	1.87 ± 0.33			Post1	Post2	−2.12	0.237
							Post3	−0.65	0.966
		Post3	1.84 ± 0.32				Post4	0.42	0.993
						Post2	Post3	2.02	0.282
		Post4	1.81 ± 0.30				Post4	2.50	0.117
						Post3	Post4	1.59	0.512
	RI	Pre	0.73 ± 0.04	2.87	0.042	Pre	Post1	−0.73	0.949
							Post2	−2.69	0.080
		Post1	0.73 ± 0.05				Post3	−2.30	0.172
							Post4	−1.23	0.736
		Post2	0.75 ± 0.04			Post1	Post2	−2.46	0.127
							Post3	−1.21	0.744
		Post3	0.74 ± 0.04				Post4	−0.21	1.000
						Post2	Post3	1.48	0.584
		Post4	0.74 ± 0.04				Post4	1.99	0.295
						Post3	Post4	1.22	0.742
AB	PI	Pre	1.88 ± 0.26	0.38	0.763	Pre	Post1	−0.76	0.939
							Post2	−0.84	0.915
		Post1	1.91 ± 0.39				Post3	−0.28	0.999
							Post4	−0.58	0.977
		Post2	1.91 ± 0.36			Post1	Post2	−0.09	1.000
							Post3	0.68	0.958
		Post3	1.89 ± 0.32				Post4	0.28	0.999
						Post2	Post3	0.99	0.857
		Post4	1.90 ± 0.31				Post4	0.46	0.990

					Post3	Post4	–0.44	0.992
RI	Pre	0.74 ± 0.03	0.32	0.802	Pre	Post1	–0.47	0.989
						Post2	–0.44	0.992
	Post1	0.75 ± 0.05				Post3	0.17	1.000
						Post4	–0.63	0.969
	Post2	0.75 ± 0.04			Post1	Post2	0.11	1.000
						Post3	0.98	0.860
	Post3	0.74 ± 0.04				Post4	–0.09	1.000
					Post2	Post3	1.01	0.848
	Post4	0.75 ± 0.04				Post4	–0.23	0.999
					Post3	Post4	–1.09	0.809

Abbreviations: VM, Valsalva maneuver; AB, abdominal bracing; PI, pulsatility index; RI, resistive index; SD, standard deviation; * $p < 0.05$.

Table S4. Comparison of vascular hemodynamic responses (carotid pulse wave velocity, vessel diameters, heart–finger pulse wave velocity) during the Valsalva maneuver and abdominal bracing.

Group	Parameter		Variable	Mean ± SD	$Mean_{diff} \pm SE_{diff}$	t	*p*
VM	Carotid PWV		Pre	4.61 ± 0.66	0.038 ± 0.134	0.58	0.566
			Post	4.53 ± 0.54			
	D_{min}		Pre	0.65 ± 0.06	–0.012 ± 0.005	–2.33	0.027*
			Post	0.66 ± 0.06			
	D_{max}		Pre	0.71 ± 0.06	–0.016 ± 0.004	–3.68	< 0.001*
			Post	0.73 ± 0.07			
	Heart-finger PWV	Lt	Pre	424.81 ± 22.00	0.003 ± 0.559	–0.01	0.995
			Post	424.81 ± 23.11			
		Rt	Pre	425.54 ± 22.08	0.343 ± 0.540	0.64	0.530
			Post	425.20 ± 22.82			
AB	Carotid PWV		Pre	4.59 ± 0.48	0.039 ± 0.129	0.30	0.768
			Post	4.55 ± 0.54			
	D_{min}		Pre	0.65 ± 0.05	–0.009 ± 0.004	–2.91	0.007*
			Post	0.66 ± 0.05			
	D_{max}		Pre	0.72 ± 0.06	–0.014 ± 0.004	–4.06	< 0.001*
			Post	0.73 ± 0.05			
	Heart–finger PWV	Lt	Pre	424.96 ± 23.51	–2.856 ± 0.999	–2.35	0.026*
			Post	427.31 ± 23.78			
		Rt	Pre	426.62 ± 23.98	1.797 ± 0.654	–2.75	0.010*
			Post	428.42 ± 24.38			

Abbreviations: VM, Valsalva maneuver; AB, abdominal bracing; PWV, pulse wave velocity; D_{min}, minimum diameter; D_{max}, maximum diameter; Lt, left; Rt, right; SD, standard deviation; SE_{diff}, standard error differences; *$p < 0.05$.

Table S5. Comparison of cerebral hemodynamic responses (regional oxygen saturation and oxyhemoglobin) during the Valsalva maneuver and abdominal bracing.

			Repeated-measures comparison				Post hoc comparison (Tukey)			
Group	Parameter		Variable	Mean ± SD	F	*p*	Repeated-measures factor		t	p_{Tukey}
VM	rSO_2	Lt	Pre	45.37 ± 13.09	5.70	0.005*	Pre	Post1	3.26	0.023*
								Post2	1.84	0.374
			Post1	41.12 ± 13.64				Post3	1.77	0.412
								Post4	1.95	0.314
			Post2	44.24 ± 14.03			Post1	Post2	–2.59	0.101
								Post3	–2.08	0.257
			Post3	43.61 ± 14.90				Post4	–2.56	0.106
							Post2	Post3	1.05	0.832

			Post4	43.74 ± 14.27				Post4	0.86	0.908
							Post3	Post4	−0.36	0.996
			Pre	48.46 ± 13.78			Pre	Post1	4.06	0.003*
								Post2	1.57	0.528
			Post1	43.02 ± 14.91				Post3	1.35	0.662
								Post4	1.91	0.334
		Rt	Post2	47.68 ± 13.88	10.99	<0.001*	Post1	Post2	−3.23	0.025*
								Post3	−3.40	0.016*
			Post3	47.63 ± 13.82				Post4	−3.53	0.012*
							Post2	Post3	0.10	1.000
			Post4	47.86 ± 13.38				Post4	−0.49	0.987
							Post3	Post4	−0.51	0.986
			Pre	$0.31 \times 10^{-3} \pm 0.05$			Pre	Post1	−2.34	0.163
								Post2	−0.46	0.990
			Post1	$8.85 \times 10^{-3} \pm 0.05$				Post3	−0.35	0.996
								Post4	0.09	1.000
		Lt	Post2	$1.66 \times 10^{-3} \pm 0.05$	4.67	0.012*	Post1	Post2	3.53	0.012*
								Post3	3.22	0.026*
			Post3	$1.34 \times 10^{-3} \pm 0.05$				Post4	3.43	0.016*
							Post2	Post3	0.35	0.997
			Post4	$0.07 \times 10^{-3} \pm 0.05$				Post4	1.43	0.617
	HbO						Post3	Post4	0.98	0.860
			Pre	$-11.75 \times 10^{-3} \pm 0.04$			Pre	Post1	−0.90	0.894
								Post2	−0.09	1.000
			Post1	$-9.40 \times 10^{-3} \pm 0.04$				Post3	0.75	0.942
								Post4	1.44	0.606
		Rt	Post2	$-11.56 \times 10^{-3} \pm 0.04$	1.99	0.129	Post1	Post2	1.01	0.847
								Post3	2.30	0.174
			Post3	$-13.52 \times 10^{-3} \pm 0.04$				Post4	2.37	0.154
							Post2	Post3	1.50	0.573
			Post4	$-14.32 \times 10^{-3} \pm 0.04$				Post4	2.09	0.255
							Post3	Post4	0.63	0.969
			Pre	44.32 ± 14.03			Pre	Post1	3.47	0.014*
								Post2	3.08	0.035*
			Post1	40.83 ± 15.09				Post3	3.32	0.020*
								Post4	2.61	0.097
		Lt	Post2	41.66 ± 16.26	6.15	0.001*	Post1	Post2	−0.91	0.890
								Post3	−1.93	0.328
			Post3	42.30 ± 15.39				Post4	−2.48	0.125
							Post2	Post3	−1.47	0.589
			Post4	42.60 ± 15.09				Post4	−1.24	0.730
AB	rSO_2						Post3	Post4	−0.63	0.969
			Pre	48.74 ± 13.95			Pre	Post1	3.36	0.018*
								Post2	2.80	0.065
			Post1	45.00 ± 15.48				Post3	2.39	0.149
								Post4	2.65	0.089
		Rt	Post2	46.58 ± 15.21	5.95	0.002*	Post1	Post2	−1.80	0.395
								Post3	−2.57	0.105
			Post3	47.13 ± 14.62				Post4	−1.70	0.451
							Post2	Post3	−1.71	0.444
			Post4	46.70 ± 15.32				Post4	−0.21	1.000
							Post3	Post4	0.86	0.908

HbO	Lt	Pre	$8.96 \times 10^{-3} \pm 0.05$			Pre	Post1	−2.95	0.047*
							Post2	−1.89	0.348
		Post1	$19.38 \times 10^{-3} \pm 0.05$				Post3	−0.15	1.000
							Post4	0.76	0.941
		Post2	$14.61 \times 10^{-3} \pm 0.05$	6.63	0.002*	Post1	Post2	2.39	0.149
							Post3	3.19	0.027*
		Post3	$9.38 \times 10^{-3} \pm 0.05$				Post4	3.22	0.025*
						Post2	Post3	3.01	0.041*
		Post4	$6.74 \times 10^{-3} \pm 0.05$				Post4	2.81	0.063
						Post3	Post4	2.19	0.215
	Rt	Pre	$-9.67 \times 10^{-3} \pm 0.04$			Pre	Post1	−2.35	0.161
							Post2	−1.82	0.385
		Post1	$-1.42 \times 10^{-3} \pm 0.04$				Post3	−0.63	0.969
							Post4	−0.25	0.999
		Post2	$-4.01 \times 10^{-3} \pm 0.04$	4.45	0.014*	Post1	Post2	1.48	0.584
							Post3	3.50	0.013*
		Post3	$-8.21 \times 10^{-3} \pm 0.04$				Post4	2.92	0.050
						Post2	Post3	2.98	0.044*
		Post4	$-9.07 \times 10^{-3} \pm 0.04$				Post4	1.96	0.314
						Post3	Post4	0.59	0.976

Abbreviations: VM, Valsalva maneuver; AB, abdominal bracing; rSO_2, regional oxygen saturation; HbO, oxyhemoglobin; Lt, left; Rt, right; SD, standard deviation; * $p < 0.05$.
